# Intact and bioactive PTH values are strongly correlated in kidney transplant recipients

**DOI:** 10.20945/2359-4292-2026-0021

**Published:** 2026-03-02

**Authors:** Tábata Carolina Faria Nascimento de Assis, Humberto Campos Clemente, Daniel Borges Drumond, Leandro Junior Lucca, Miguel Moyses-Neto, Francisco José Albuquerque de Paula, Elen Almeida Romão

**Affiliations:** 1 Divisão de Nefrologia, Departamento de Clínica Médica, Faculdade de Medicina de Ribeirão Preto, Universidade de São Paulo, São Paulo, SP, Brasil; 2 Faculdade de Medicina de Ribeirão Preto, Universidade de São Paulo, Ribeirão Preto, SP, Brasil; 3 Divisão de Endocrinologia e Metabologia, Departamento de Clínica Médica, Faculdade de Medicina de Ribeirão Preto, Universidade de São Paulo, Ribeirão Preto, SP, Brasil

**Keywords:** Kidney transplantation, parathyroid hormone, vascular calcification, chronic kidney disease-mineral and bone disorder

## Abstract

**Objective:**

This study aims to evaluate the correlation between parathyroid hormone measured by a second-generation assay (PTHG2) and by a third-generation assay (PTHG3), and their association with mineral and bone disorder (MBD) biochemical parameters and radiographic vascular calcification score in kidney transplant recipients (KTRs).

**Materials and methods:**

We evaluated 87 KTRs and measured PTHG2, PTHG3, biochemical profile, urinary fractional excretion of calcium (FE Ca) and phosphate, 25(OH)D_3_, and Kauppila score for vascular calcification. Statistical analysis: Non-parametric tests and logistic regression analysis were performed. The significance level was set to 5%.

**Results:**

In our population, the mean age was 54 years, the mean time after transplantation was 9.4 years (± 7.6), and the mean estimated glomerular filtration rate (GFR, calculated using the Chronic Kidney Disease Epidemiology Collaboration equation - CKD-EPI) was 59.1 mL/min/1.73 m^2^. The correlation between PTHG2 and PTHG3 was almost perfect (r = 0.99; 95% CI = 0.98-0.99) and there was no significant difference between the PTHG2/PTHG3 ratio from different KTR-CKD stages. Investigating the association among PTH and the MBD biochemical profile, there was only correlation between PTH and FE Ca (p-value 0.01).

**Conclusion:**

We concluded that there is no advantage in PTHG3 dosage over PTHG2 measurement in this population.

## INTRODUCTION

Chronic kidney disease (CKD) is a condition of high morbidity and mortality. Its prevalence reached about 9.1% of the world population and more than 2.5 million people were on renal replacement therapy in 2017 ^(1)^. In this context, mineral and bone disorders (MBD) stand out among the factors that increase CKD morbidity ^(2)^.

Kidney transplant is the best choice for treatment of end-stage CKD, as it promotes higher quality and life expectancy ^(3)^. However, mineral and bone disease may remain after renal transplantation, either due to persistent hyperparathyroidism, chronic use of corticosteroids, or progressive renal graft dysfunction ^(4)^.

From the earliest stages of CKD, there is a reduction in urinary phosphorus excretion. As compensation, there is an increase in phosphaturic mechanisms, represented by FGF-23 and parathyroid hormone (PTH). PTH 1-84 is an 84 amino acid polypeptide secreted by parathyroid principal cells under hypocalcemia stimulus - the initial sequence of amino acids being the amino-terminal fragment and the last sequence, the carboxy-terminal fragment ^(5)^.

The first 34 amino acids of the amino-terminal portion are involved in the activation of the PTH1R receptor. Therefore, PTH 1-34 corresponds to the biologically active fragment, responsible for its hormonal action in bones and kidneys ^(5)^.

Under hypercalcemia, PTH is cleaved into several parts in the parathyroid (before its secretion) and in the liver (in peripheral metabolism): the amino-terminal fragments are rapidly degraded and the carboxy-terminal fragments remain in the bloodstream, from where it is slowly filtered by the kidneys ^(6)^. These carboxy-terminal fragments usually accumulate in renal failure. Furthermore, the inflammatory state of CKD increases oxidative stress and, consequently, post-translational modifications of the PTH molecule, such as the oxidation of methionine residues at positions 8 and 18 ^(7)^. Thus, one may conclude that PTH measurement can be extremely challenging.

Non-bioactive presentations often mentioned in previous studies are the PTH 7-84 fragment and the oxidized PTH ^(7,8)^, which curiously were not detected by high-resolution mass spectrometry. This fact, apart from possible methodological disparities, raises doubts about the real role of these presentations *in vivo* ^(6)^.

Over the past few decades, increasingly sensitive tests have been developed to measure PTH levels. The most commercially available is the second-generation test (PTHG2), which has the inconvenience of also measuring non-bioactive fragments of PTH. Newer third-generation methods (PTHG3) have emerged to address these technical problems, with final values approximately reduced to half of the PTHG2 measurements ^(9)^.

Despite the variety of studies about PTH measurement methods in patients with CKD, few studies have been conducted to evaluate these different methodologies in kidney transplant recipients (KTRs). Our study aims to evaluate the correlation between PTHG2 and PTHG3 to clarify whether there would be an advantage in PTHG3 dosing in this population. Furthermore, we intend to investigate whether PTHG2 and PTHG3 have a stronger association with vascular calcification (Kauppila score) and some BMD parameters after kidney transplantation. We hypothesized that intact and bioactive PTH values are also correlated in KTRs.

## MATERIALS AND METHODS

### Study population

This is a cross-sectional, convenience sampling, single center study. We included adults who had received at least 90 days previously, a single renal transplant between November 2016 and October 2018. We excluded patients with inadequate biological samples or with clinical complications at the time of recruitment, such as rejection, infectious diseases, and neoplasms. All patients were classified into 5 stages of CKD, according to KDIGO guidelines ^(3)^. The clinical data were collected from medical records and interviews.

All patients gave written informed consent. This study was approved by the Research Ethics Committee of the Clinical Hospital of Ribeirão Preto, University of São Paulo in Ribeirão Preto, Brazil (*Hospital das Clínicas da Faculdade de Medicina da USP de Ribeirão Preto* - HCFMRP-USP), No. 1.746.953, in 2016.

### Laboratory parameters

Fasting blood samples were collected for measurement of total calcium, albumin, phosphorus, and alkaline phosphatase. In urine samples, we measured creatinine, calcium, and phosphorus. The tests were performed using the enzymatic method (WIENER^®)^, according to standard laboratory protocols. The urinary fractional excretion of calcium (FE Ca) and phosphate were calculated with the usual equations.

The 25-hydroxyvitamin D [25(OH)D_3_] was evaluated by chemiluminescence using the LIAISON^®^ test. PTHG2 measurements were performed with the LIAISON^®^ N-TACT^®^ kit (reference value range: 14.5-87.1 pg/mL), while PTHG3 was evaluated by the LIAISON^®^ 1-84 PTH assay (reference value range: 6.7-38.8 pg/mL), both from DiaSorin (Saluggia, Italy). Samples for PTHG2 and PTHG3 determinations were centrifugated and subsequently frozen at -70 °C for later duplicate analysis.

### Radiographic parameters

The assessment of vascular calcification was performed using the Kauppila score. This score indicates a greater cardiovascular risk (a positive index) in the presence of seven or more points of calcification in the abdominal aorta parallel to the L1-L4 vertebrae on a lateral abdominal x-ray, on a scale that ranges from 0 to 24 ^(10)^.

Two experienced radiologists classified the radiographs according to the Kauppila score. Agreement between evaluators regarding the presence of vascular calcification was analyzed, with a Kappa coefficient of 0.91.

### Statistical analysis

Epidemiological and clinical data are presented as mean (standard deviation), median (minimal - maximal values), or percentage. We used LibreOffice Writer version 7.1.6.2, LibreOffice Calc version 7.1.6.2, and R for Windows version 4.0.2. The significance level was set to 5%.

To investigate whether there is PTHG2 retention with CKD progression, the Kruskal-Wallis test was performed to analyze the PTHG2/PTHG3 ratio for each CKD stage. Correlation studies were performed using Spearman’s rank correlation coefficient.

Finally, a logistic regression model was adjusted for the Kauppila calcification score and each PTH method.

## RESULTS

### Patient characteristics

A total of 87 patients were evaluated. Demographic, clinical, and laboratory data are presented in **Tables 1** and **2**. The mean time after transplantation was 9.4 years (± 7.6). Induction of immunosuppression was performed with thymoglobulin and basiliximab in 49.43% and 48.28% of cases, respectively. Current immunosuppression consisted mainly of prednisone, tacrolimus, and mycophenolate sodium. Regarding specific medications for CKD-MBD, 21.84% used cholecalciferol or ergocalciferol, 12.6% used cinacalcet, and 3.45% used calcitriol. Finally, **Table 3** describes PTHG2 and PTHG3 values according to CKD stage.

**Table 1:** Demographic and clinical data

Variables	All patients
Age (years), mean (standard deviation)	54 (13.0)
Male, No. (%)	53 (60.92)
Parathyroidectomy, No. (%)	4 (4.5)
Modality of Dialysis	
Haemodialysis, No. (%)	69 (79.3)
Peritoneal dialysis, No. (%)	3 (3.4)
Both, no. (%)	14 (16.1)
None, no. (%)	1 (1.2)
Chronic kidney disease aetiology	
Hypertension, No. (%)	22 (25.29)
Diabetes mellitus, No. (%)	14 (16.09)
Polycystic kidney disease, No. (%)	3 (3.45)
Glomerulopathy, No. (%)	21 (24.14)
Urinary obstruction, No. (%)	3 (3.45)
Unknown aetiology, No. (%)	24 (27.7)
Donor	
Living, No. (%)	19 (21.84)
Deceased, No. (%)	68 (78.2)
Retransplant, No. (%)	4 (4.6)

**Table 2:** Laboratory data

Variable	No.	Minimum	Median	Maximum
CKD-EPI (mL/min/1,73 m^2)^	87	14.0	59.0	125.0
PTHG2 (pg/mL)	87	10.6	119.0	739.0
PTHG3 (pg/mL)	87	5.9	46.2	246.0
25(OH)D_3_ (ng/mL)	85	5.0	23.8	61.0
ALP (U/L)	87	84.5	175.0	554.6
Phosphorus (mg/dL)	87	1.8	3.4	6.1
Total Calcium (mg/dL)	87	7.3	9.8	12.6
Albumin (g/dL)	87	3.6	4.3	4.8
FE Ca (%)	87	0.0	0.6	7.8
FE P (%)	85	2.0	17.7	41.8

ALP: alkaline phosphatase; FE Ca: fractional excretion of calcium; FE P: fractional excretion of phosphate; CV: coefficient of variation.

**Table 3:** Descriptive data of PTHG2 and PTHG3 according to CKD stage. Correlation between PTHG2 and PTHG3 for the total sample and for each CKD stage

CKD	*n*	PTH	Median	Minimum-Maximum	p-Value^*^	Spearman (rho)#	95% CI
Total	87	G2	119.0	10.6-739	<0.001	0.99	0.98-0.99
		G3	46.2	5.9-246			
1	9	G2	119	38.3-252	0.004	0.93	
		G3	46.2	14-82			
2	34	G2	102.6	10.6-739	<0.001	0.99	
		G3	40.6	5.9-246			
3A	19	G2	143.0	36.3-493	<0.001	0.99	
		G3	52.3	16.1-161			
3B	15	G2	78.9	41.3-168	<0.001	0.94	
		G3	30.9	16.6-65			
4	10	G2	192.5	123-694	0.002	0.96	
		G3	70.4	51.1-229			

CV: coefficient of variation; CI: confidence interval.

* Wilcoxon test p-Values to verify the difference between PTHG2 and PTHG3 for the total sample and for each CKD stage.

# Spearman correlation coefficient (rho) between PTHG2 and PTHG3 for total sample and for each CKD stage.

### Primary endpoints

**Figure 1:**
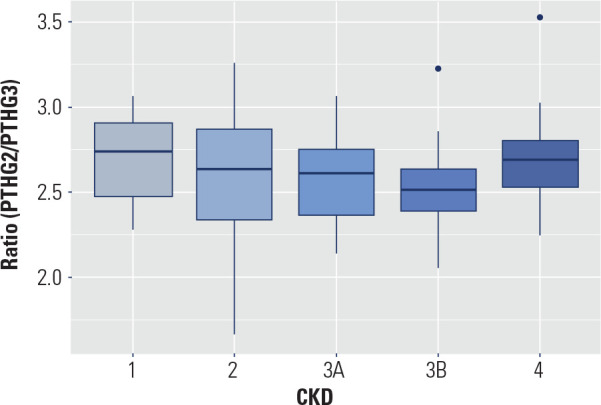
PTHG2/PTHG3 ratio according to CKD stage *box plot*.

In all CKD stages, PTHG2 values were higher than PTHG3 values. PTHG2 was approximately 2.6 times greater than PTHG3 **(Table 3)**. At the 5% significance level, there is no evidence that the PTHG2/PTHG3 ratio changes between CKD stages (Kruskal-Wallis test p-value 0.37). These data are best visualized in the box plot of **Figure 1**.

**Figure 2:**
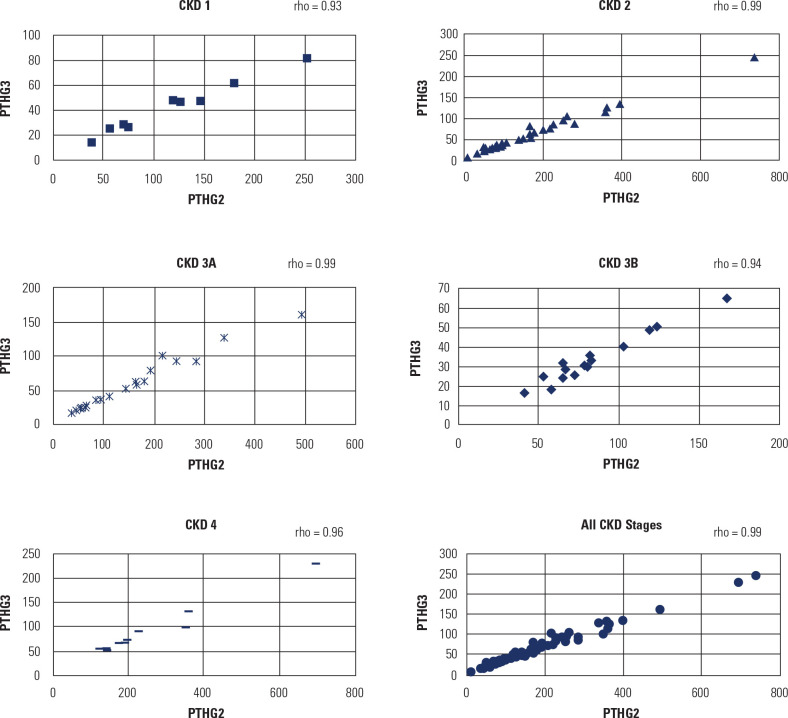
Dispersion diagram. Spearman correlation (rho) between PTHG2 and PTHG3 according to stage of kidney disease.

**Table 3** presents the Spearman correlation coefficients between PTHG2 and PTHG3 for the complete sample and for each CKD stage. As seen in this table and in the scatterplots shown in **Figure 2**, these two variables are highly correlated. The minimum value was 0.93 for CKD stage 1, 0.94 for stage 3B, and 0.96 for stage 4, which means that the correlation remains high even at the most severe levels of the renal disease.

### Secondary endpoints

Considering the interference of drugs and parathyroidectomy in bone metabolism, we excluded patients who were using calcitriol, cholecalciferol, ergocalciferol, and cinacalcet from the analysis of the secondary endpoints, as well as patients who had undergone parathyroidectomy. Thus, 58 patients were studied.

**Table 4** shows Spearman correlation coefficient values between some laboratory variables and PTHG2 and PTHG3, as well as the corresponding p-values. At the 5% significance level, there is evidence of a negative association between PTH (regardless of the method) and glomerular filtration rate (GFR) and a positive association between PTH (regardless of the method) and FE Ca. There was no correlation between PTH values (regardless of the method).

**Table 4:** Spearman correlation coefficients (rho) between some laboratory variables and PTHG2 and PTHG3 - 58 patients

	PTHG2			PTHG3	
Variable	rho	p-Value		rho	p-Value
**CKD-EPI**	**-0.27**	**0.037**		**-0.30**	**0.024**
25(OH)D_3_	-0.15	0.275		-0.15	0.263
ALP	0.02	0.888		0.03	0.834
Phosphorus	-0.02	0.913		-0.02	0.912
Total Calcium	0.06	0.633		0.05	0.703
**FE Ca**	**0.33**	**0.011**		**0.33**	**0.011**
FE P	0.15	0.282		0.18	0.176

ALP: alkaline phosphatase; FE Ca: fractional excretion of calcium; FE P: fractional excretion of phosphate.

To verify whether there is an effect of PTHG2 and PTHG3 on the Kauppila calcification score **(Table A.1)**, a logistic regression model was adjusted for each PTH method. **Table 5** shows that at 5% significance level, all models are well adjusted (p-values for the Hosmer and Lemeshow test ≥ 0.57) and that there is no evidence of an effect of PTHG2 and PTHG3 on calcification scores (p-values ≥ 0.68).

**Table 5:** Estimates and p-values related to the logistic regression and the Hosmer and Lemeshow test (HL)

Calcification Score	PTHG2				PTHG3		
	Estimate	p-value	p-value HL test		Estimate	p-value	p-value HL test
Kauppila	0.0006	0.818	0.567		0.0033	0.677	0.764

## DISCUSSION

The determination of PTH by both PTHG2 and PTHG3 includes the use of two antibodies in “sandwich”: the first antibody captures the carboxy-terminal fragment of PTH (usually amino acids 39-84), while the second antibody selects and quantifies, among the captured molecules, those with the amino-terminal fragment - being an intermediate amino acid sequence in PTHG2 tests (e.g., 12-24, 26-32, 12-18, 13-34, etc.) and an initial sequence in PTHG3 tests (e.g., 1-4) ^(6,8,11)^.

As expected, PTHG2 does not identify the beginning of the amino-terminal sequence and may quantify undesirable PTH fragments which lost the first amino acids. As stated previously, PTH 1-34 is the biologically active fragment ^(5)^.

In contrast, PTHG3 detects the first and the last amino acids of the molecule, identifying the “whole” PTH. However, PTHG3 cross-reacts with forms of PTH 1-84 that have undergone post-translational changes, such as such as the oxidation of methionine residues at positions 8 and 18 and the phosphorylation of serine 17. The latter represents a molecule hyper-secreted in parathyroid neoplasms, corresponding to 15% of the final PTHG3 measurement in patients with CKD ^(6,7,12,13)^.

As a primary endpoint, our study evaluated the correlation between PTHG2 (intact PTH) and PTHG3 (bioactive PTH) levels in KTRs. A very strong correlation (r = 0.99; 95% CI = 0.98 - 0.99) was found between PTHG2 and PTHG3 levels, with the value of PTHG2 being about 2.6 times the PTHG3 measurement, a higher ratio than found in previous studies ^(9,13)^.

Our study agrees with data previously found in chronic renal volunteers. Einbinder and cols., in a study that evaluated 98 patients with non-dialytic CKD in stages 3, 4, and 5, found a strong correlation (r = 0.963, p-value < 0.01) between PTHG2 and PTHG3 values ^(14)^. O’Flaherty and cols., in a study comprising 140 pre-dialysis volunteers, found a correlation in patients with end-stage CKD (r = 0.98, p-value < 0.01) and in patients with better CKD stages (r = 0.96, p-value < 0.01) ^(15)^. Gannagé-Yared and cols., in a study comprising 92 patients on hemodialysis, found a significant correlation between PTHG2 and PTHG3 (r = 0.923, p-value < 0.01) ^(13)^. Melamed and cols., in a study that evaluated 515 patients on hemodialysis and peritoneal dialysis, found a very strong correlation between PTHG2 and PTHG3 (r = 0.99, p-value < 0.01) ^(9)^.

The PTHG2/PTHG3 ratio did not vary significantly between CKD stages (p-value 0.37). These data suggest that, despite the retention of unwanted PTH fragments, a proportional increase in PTHG2 relative to PTHG3 was not found as the GFR was reduced. Different results were found by O’Flaherty and cols., who observed 18% lower PTHG3 values in stages 1-4 and 40% lower PTHG3 values in stage 5 when compared to PTHG2 ^(15)^.

In the present study, the negative correlation between PTH and GFR was expected since this variable determines the severity of the renal disease. Except for the FE Ca, there were no correlations between PTHG2 or PTHG3 levels and other MBD parameters and Kauppila calcification score. Probably the serum calcium levels remained stable because of a greater urinary calcium excretion as PTH increases. It is noteworthy that, although significant, the correlation between PTH and FE Ca can be considered weak in both cases (the highest absolute value was 0.33).

In the pre-dialysis population, Einbinder and cols. found significant correlations between both PTH measurement methods and calcium, phosphorus, and 25(OH)D3 values, while O’Flaherty and cols. found only correlation between PTHG3 (bioactive PTH; 1-84) and calcium and phosphorus levels ^(14,15)^.

In the dialysis population, Gannagé-Yared and cols. found a correlation only between PTH (regardless of the method) and alkaline phosphatase and type I collagen degradation products ^(13)^. In contrast, the CHOICE study found a negative correlation with calcium and a positive correlation with phosphorus between PTHG3 (bioactive PTH; 1-84) and unwanted PTH fragments (represented by the PTHG2-PTHG3 difference). In the latter study, mortality was associated only with PTHG3 ^(9)^.

To our knowledge, the only study that evaluated the correlation between PTHG2 and PTHG3 in transplanted patients was carried out by Tan and cols. These authors evaluated 83 patients: 44 pre-dialysis, 15 on hemodialysis, 15 on peritoneal dialysis, and 9 after renal transplantation. PTHG2 and PTHG3 had a good correlation, and PTHG3 (PTH 1-84) showed a positive association with phosphate and alkaline phosphatase ^(16)^. However, unlike our study, all KTRs included were already in end-stage CKD.

Our study has several limitations, such as the small number of patients, the single-center and convenience sampling, the cross-sectional design, and the absence of multivariate analyses. These limitations must be acknowledged, as they may restrict the generalizability of the findings.

In summary, based on our data, it is possible to establish a very strong correlation between levels of PTHG2 and PTHG3 in KTRs. Therefore, our study suggests that there is no advantage in PTHG3 dosage over PTHG2 measurement. However, apart from the FE Ca, no correlations have been established between PTH, several MBD parameters, and Kauppila calcification score. Further longitudinal studies are needed.

## Data Availability

datasets related to this article will be available upon request to the corresponding author.

## APPENDICE

**Table A.1:** Descriptive measures of PTHG2 and PTHG3 by Kauppila Score - 58 patients

Index	n	PTH	Mean	Standard deviation	Minimum	Median	Maximum
Negative	35	G2	143.1	134.8	10.6	85.1	739
		G3	53.1	45.3	5.9	35.7	246
Positive	11	G2	153.2	95.2	36.3	123.0	360
		G3	59.2	32.3	16.1	50.5	114
NA	12	G2	148.3	86.9	34.1	154.0	283
		G3	56.7	30.4	15.0	56.5	93
